# MotifCluster: an interactive online tool for clustering and visualizing sequences using shared motifs

**DOI:** 10.1186/gb-2008-9-8-r128

**Published:** 2008-08-15

**Authors:** Micah Hamady, Jeremy Widmann, Shelley D Copley, Rob Knight

**Affiliations:** 1 Department of Computer Science, University of Colorado, Boulder, CO 80309, USA; 2 Department of Chemistry and Biochemistry, University of Colorado, Boulder, CO 80309, USA; 3 Department of Molecular, Cellular and Developmental Biology and Cooperative Institute for Research in Environmental Sciences (CIRES), University of Colorado, Boulder, CO 80309, USA

## Abstract

MotifCluster finds related motifs in a set of sequences, and clusters the sequences into families using the motifs they contain. MotifCluster, at , lets users test whether proteins are related, cluster sequences by shared conserved motifs, and visualize motifs mapped onto trees, sequences and three-dimensional structures. We demonstrate MotifCluster's accuracy using gold-standard protein superfamilies; using recommended settings, families were assigned to the correct superfamilies with 0.17% false positive and no false negative assignments.

## Abstract 2

MotifCluster finds related motifs in a set of sequences and clusters the sequences into families using the motifs they contain.

## Rationale

Detection of evolutionary relationships between very distantly related protein families is important for efforts to assign functions to newly identified proteins, as well as to understand the evolutionary mechanisms by which new functions have emerged. Pairwise sequence identities between proteins in distantly related families are often statistically insignificant. Algorithms such as COMPASS [1] that evaluate relationships between profiles representative of protein families are perhaps the most powerful method for identification of distant sequence relationships, although iterative BLAST approaches, such as PSI-BLAST [2] and SHOTGUN [3], are also valuable. Identification of evolutionary relationships between protein families and superfamilies sets the stage for analysis of the sequence changes that led to the distinctive structural and functional characteristics of protein families. In many enzyme superfamilies, the ability to catalyze an ancestral catalytic step has been retained, while additional steps have been added before or after the ancestral step. For example, in the enolase superfamily, abstraction of a proton from a position alpha to a carbonyl is the conserved catalytic step; the fate of the resulting enolate intermediate varies in different families according to the disposition of catalytic groups in the active site [4].

Identification of short, highly conserved sequences, known as motifs, in proteins provides important insights into the regions of proteins that have been conserved within a superfamily or suprafamily, as well as those that have diverged in specific families. Consideration of these motifs in conjunction with mechanistic and structural information can provide a picture of the sequence changes that led to acquisition of new catalytic capabilities. Two motif-finding algorithms, MEME (Multiple EM for motif elicitation) [5] and the Gibbs Sampler [6], are in widespread use. MEME identifies motifs by searching for a set of short, conserved sequences (motifs) in a set of longer, less conserved sequences. MEME assumes that each of the sequences in the input set contains at least one motif. The Gibbs Sampler works by searching for a predefined number of motifs with minimum and maximum lengths. A background probability model for chance matches based on amino acid occurrences is determined from the input set of sequences. Motifs are discovered by searching for regions in the set of sequences that do not fit this background probability model. Both algorithms report motifs present in subsets of a user-provided set of sequences, along with statistical information regarding the significance of each motif in the entire set as well as within a particular sequence.

A drawback of these algorithms is that the results depend on the order of sequences provided in the input set in an unpredictable way. Clustering sets of sequences based upon visual analysis of motifs, as in [7], is both subjective and time-consuming, as it requires re-ordering of the input set. Furthermore, motifs are presented solely in terms of primary sequence; mapping of motifs onto structures, which is critical for recognizing the roles played by specific motifs, requires additional manipulation.

In this paper, we present a new online tool, MotifCluster, that clusters input sequences according to the presence or absence of user-supplied motifs. MotifCluster uses any of six different distance metrics. Some of these metrics group sequences that contain the same motifs in the same order, and others look solely at which motifs are shared. The ability to take order into account is critical in some cases, because motifs may need to be in the context of a specific structural context to have biological activity. However, domain shuffling and circular permutation of sequences are not uncommon, so it can also be important to recognize the occurrence of shared motifs in an unusual order. Longer or more highly significant motifs can be given more weight than shorter or less significant motifs, or all motifs can be treated equally. Sequences can also be labeled with user-defined designations, such as family assignment, and the associations between families and motifs can then be used to explore functional relationships. In addition to clustering input sequences according to the motifs they contain, MotifCluster automatically maps motifs onto the structures of all proteins in the set for which structural information is available, providing an immediate visual assessment of the location of each motif. MotifCluster can be used online from the URL provided in the abstract, which also links to documentation and a downloadable version.

## Key features

MotifCluster allows sequences to be clustered according to their shared motifs in several ways, and facilitates identification of relationships between groups of sequences that share specific motifs. MotifCluster provides methods for testing whether different sequence families are related to one another, whether the motifs are meaningful in the context of the structures corresponding to each sequence (when available), and whether the patterns of motifs identified are consistent with standard phylogenetic analysis. The latter feature is particularly important, as standard phylogenetic analysis becomes difficult when sequences are highly diverse because alignments become unreliable below about 30% sequence identity [8]. Brief summaries of MotifCluster functionality and that of several tools related to it can be found in Table 1.

**Table 1:** Summary of key features of MotifCluster and a selection of other programs that perform clustering of motifs or remote homology detection

Strategy	Program	Overview of program	Publication
Clustering proteins by motifs they contain	MotifCluster	Takes aligned or unaligned protein and nucleotide sequences and a MEME file showing motifs; allows clustering of the sequences according to the motifs they contain, and visualization of the motifs on the aligned and unaligned sequences and three-dimensional structures	This article
Clustering of transcription factor binding sites (in DNA)	MCAST	Takes list of transcription factor binding sites as input: uses hidden Markov models to find *cis*-regulatory modules in DNA	[21]
	Cluster-Buster	Takes list of transcription factor binding sites as input: uses Forward algorithm and expected uniform distribution to find motif co-occurrence in DNA	[22]
	ClusterDraw	Takes list of transcription factor binding sites as input: uses r-scan algorithm and sweep over parameter values to visualize significant clusters as peaks on the DNA sequence	[23]
	COMET	Calculates significance of collection of position-specific score matrices that appear in order: can apply to DNA or protein, in principle	[24]
	PEAKS	Calculates significance of collection of transcription factor binding sites that appear at specified distance from transcription start site or other feature in the DNA	[25]
	CompMoby	Aligns all pairs of motifs that appear significant in different promoters, then groups these into clusters using the CAST algorithm. DNA-specific	[26]
	CREME	Identifies groups of DNA motifs that co-occur significantly within a defined distance using both order-dependent and order-independent models	[27]
	PHYLOCLUS	Uses Bayesian method to find clusters of evolutionarily conserved DNA motifs that appear in different promoters.	[28]
	INCLUSive	Clusters genes based on microarray analysis: feeds promoters to Gibbs sampler to find DNA motifs overrepresented in each cluster	[29]
Identifying kernels for SVMs*	SVM kernels	Introduces kernels based on k-word occurrences and best BLAST hit for SVM clustering: does not focus on conserved motifs	[30]
	WCM (word correlation matrices)	Introduces k-word kernel for SVM clustering based on correlations in appearance of pairs of k-words: does not focus on conserved motifs.	[31]
	ODH (oligomer distance histograms)	Introduces new kernel for SVM clustering based on histograms of distances between all words in protein: does not focus on conserved motifs	[32]
Iterative BLAST	Shotgun	BLAST-based approach for identifying remote homologs by iterative searches: not motif-based	[3]
	DivergentSet	Among other features, can perform BLAST and PSI-BLAST versions of Shotgun and choose representative sequences of each group: not motif-based	[20]
	Cascade PSI-BLAST	Performs iterative steps of PSI-BLAST, otherwise like Shotgun: not motif-based.	[33]
	ProClust	Performs graph-based connection of proteins based on pairwise sequence similarity: not motif based	[34]
k-word clustering	CD-Hit	Clusters proteins based on shared segments of overall sequence, not by motifs already known to be significant	[35]
Profile-profile alignment	COMPASS	Performs profile-profile alignments for remote homology detection: assesses statistical significance matches in the profiles overall, rather than specifically using shared motifs	[1]
Clustering of motifs	STAMP	Aligns motifs with one another so that relationships among motifs can be detected; performs many other tasks for promoter characterization, but specific to promoters	[36]
	TAMO	Performs many functions for *cis*-regulatory analysis: is able to cluster DNA motifs with one another	[37]
	SOMBRERO	Aligns and clusters DNA motifs with one another to improve transcription factor binding site searches	[38]
Identification of functions in labeled structures	FunClust	Takes set of three-dimensional structures with annotated functions; identifies three-dimensional motif fragments that are common to the structures with each function.	[39]

*SVMs are support vector machines, a common machine learning approach to pattern classification. A kernel is a function that calculates the inner product of all pairs of input vectors in an abstract space, which is an important step in the process and affects the clustering.

Several reports are generated after uploading a set of aligned or unaligned sequences, motif information, and, optionally, mappings that relate sequence identifiers (IDs) to known gene families. In these reports, each motif is assigned a unique style (color and font display, for example, bold or italic) that is used consistently throughout the displays. Each report format can be selected from a drop down list on the search results page.

### Displaying motifs on trees

The first four report formats display motifs, either including or excluding the rest of the sequence, on a tree based either on the sequences or the motifs. These reports are important for establishing whether a particular motif fits the overall phylogenetic pattern, or has evolved convergently in different lineages, and can be especially useful for establishing relationships between sequences that are too diverse for construction of phylogenetic trees using standard methods. They are also important for visualizing where the motifs occur in the sequences, which can be important for detecting domain shuffling. These four report formats are described below.

#### Motifs on motif-based tree

This tree is built using a matrix of distances calculated from the motif-based alignment. The metric used to build this tree reflects similarities only among the motifs (and not in the rest of the sequences). The color-coded motifs and location information are displayed, along with links to available Protein Data Bank (PDB) structures. The PDB links allow the user to view the motifs found in a particular sequence on the corresponding structure using PyMol [9]. This format is especially useful for visually evaluating whether the clustering method chosen groups the motifs together in an intuitively reasonable way, and for checking whether motifs are shuffled or circularly permuted.

#### Sequences on motif-based tree

Similar to the 'Motifs on motif-based tree' format above, except that the full sequences are shown (rather than just the sequences of the motifs). This format is useful for deciding whether there are extended regions of conservation around the motifs in specific groups of sequences, and like the format above, for identifying domain shuffling or circular permutation of motifs.

#### Motifs on sequence-based tree (full-length)

Similar to the 'Sequences on motif-based tree' format above, except that a phylogenetic tree is generated using MUSCLE (Multiple sequence comparison by log-expectation; with default parameters) based on similarities among the full-length sequences. Motifs are highlighted and displayed as for the 'Motifs on motif-based tree' format. This display is useful for testing whether the pattern of motif conservation follows the overall conservation of the sequences: if motifs have not evolved convergently and the sequences are sufficiently closely related to retain phylogenetic signal, the tree built using the motifs will be approximately the same as the tree built using the sequences, and, in both cases, large clades of sequences containing the motifs should be observed. Alternatively, if the sequences are so highly diverged that phylogenetic reconstruction is unreliable (the so-called 'twilight zone' below 30% conservation [8]), the motif tree may cluster family members together when the sequence tree is uninformative.

#### Motifs on sequence-based tree (motif regions only)

Similar to 'Sequences on motif-based tree' and 'Motifs on sequence-based tree (full-length)' formats above: displays only the sequences of the motifs on the tree built using the full-length sequences.

### Identifying which sequences contain particular motifs

The next group of reports shows which sequences contain each motif. These reports are useful for evaluating which motifs are meaningful, and which tend to occur together in the same sequences.

#### Statistics by sequence

Displays a table of motifs and associated *P*-values, grouped by sequence. For each sequence, motifs are displayed in order of decreasing significance (ascending *P*-value). A display underneath the sequence indicates conservation: positions annotated with asterisks match the motif consensus at positions that are not highly conserved (according to a user-defined threshold, which is set to 90% by default); positions annotated with gray shaded + sighs match the consensus at positions that are highly conserved, and red positions are mismatches at positions that are highly conserved. This format is especially useful for finding systematic differences that may be functionally important within motifs. For example, single amino acid changes in a motif conserved in a superfamily may be related to divergence in function in a particular family.

#### Statistics by motif

Displays a table of motifs and associated *P*-values, grouped by motif. For each motif, an alignment of the motif regions is displayed, with the majority consensus of the motif displayed above the alignment. Highly conserved columns (determined by the 'conservation threshold' parameter) in the consensus motif are colored. Positions within individual motifs are highlighted in grey if they match the consensus sequence. Like the 'Statistics by sequence' format, this format is useful for finding sequence changes that are potentially associated with functional changes.

### Exploratory analyses

The final group of reports provides tools for exploratory analysis.

#### Highlight alignment

Displays an interactive form allowing the user to select specific motifs to highlight in the alignment. This format is useful for assisting in decisions about which motifs are likely to be real and which are false positives, and reduces the visual complexity in the motif- and sequence-based tree formats by allowing the user to focus on specific motifs of interest.

#### Network view

Displays all sequences in a network representation, with each connected component drawn as a separate network. The connected components are determined by the 'edge threshold' parameter. For example, if this parameter is 1, each connected component consists of all the sequences that share at least one motif with any other sequence in the connected component. If the parameter is 2, all sequences in a connected component must share at least two motifs with at least one other sequence in the same connected component. The list of IDs for the sequences in each connected component is displayed, and the actual sequences in each connected component can be downloaded as a FASTA file.

#### Supported motif formats

We currently support motifs generated using either MEME [5] or the Gibbs sampler [6] so that users can easily compare the two methods. We plan to add support for other motif definitions, including user-supplied weight matrices, and for other motif finding algorithms.

## Distance calculations and clustering

### Measuring distance between sequences using motifs

**Figure 1:**
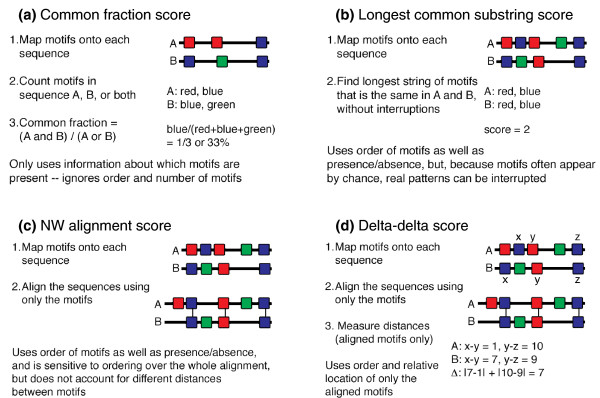
Methods for measuring distances between sequences using motif information. **(a) **Common fraction score; **(b) **Longest common substring score; **(c) **Needleman-Wunsch alignment score; **(d) **delta-delta score.

The distance between pairs of sequences based upon the motifs they contain can be calculated using several methods. The following distance measures are currently implemented in MotifCluster (Figure 1).

#### Common fraction score

The Common fraction score method (Figure 1a) calculates the fraction of motifs shared between each pair of sequences, ignoring the order in which the motifs occur in the sequence and the number of times each motif occurs.

#### Longest common substring score

The Longest common substring (LCS) score method (Figure 1b) finds the longest common substring of motifs that occurs in both sequences. Instead of using the actual motif sequences, the substring is constructed by assigning a unique character to each motif, and then using suffix trees to calculate the longest pattern of motifs that occurs in the same order in both sequences. This method does not account for differences in spacing between the motifs. The LCS score is a measure of similarity, which is converted into a distance metric in two different ways. LCS (max-actual) scores the distance as the difference between the best LCS score for any pair of sequences in the set and the LCS score for the pair of sequences under consideration. LCS (1-(actual/max)) scores the distance based on the ratio between the LCS score for the pair of sequences under consideration and the best LCS score for any pair of sequences in the set.

#### Needleman-Wunsch score

The Needleman-Wunsch (NW) score method (Figure 1c) uses the NW global pairwise alignment algorithm [10] to align the two motif strings, converted from the raw sequences to unique characters as described for LCS scores above. The method can either be unweighted (all motifs are treated equally), or weighted (highly significant motifs count for more than less significant motifs). Like the LCS score, the raw NW score is a measure of similarity. It is converted into a distance metric using the same methodology (either (max-actual) or (1-(actual/max))). Like the LCS score, the NW score takes into account the order, but not the spacing, between the motifs. Unlike the LCS score, the NW score is robust to insertions and deletions that disrupt what is otherwise a long, shared sequence of motifs.

#### Delta-delta score

The delta-delta score (Figure 1d) measures the distances between aligned motifs in each pair of sequences, and sums the differences in distances between each pair of motifs in the aligned pair of sequences. Aligned motifs with equal spacing in both sequences have a delta-delta score of zero. When motif spacing is unequal, the delta-delta score is > 0. The delta-delta score is thus a distance metric, and does not need to be converted from a similarity metric as do the LCS and NW scores.

### UPGMA clustering

The UPGMA (Unweighted pair group method with arithmetic mean) clustering algorithm [11] uses a distance matrix to find successive nested clusters by identifying the nearest neighbors at each step, then merging these neighbors. When performing motif-based clustering, we generate the distance matrix using one of the user-specified distance measures, and use this distance matrix as input into the UPGMA routine, yielding a tree that clusters the sequences according to the motifs they contain. To compare the motif-based clustering with traditional sequence-based clustering, we also generate trees using MUSCLE [12]. MUSCLE groups the sequences using the fraction of words of a specific length that are shared between the sequences, and thus estimates the overall distance between the entire sequences rather than just between the motifs.

### Graphs and connected components

We generate a weighted graph showing the relationships between all sequences in terms of the motifs they share. Vertices in the graph represent a sequence in the input set, and each edge in the graph represents a relationship in which two sequences share one or more motifs. Thus, each sequence is connected to every other sequence with which it shares at least one common motif. The weight of each edge in the graph is calculated as the number of motifs shared by each pair of sequences. Once the full weighted graph has been generated, edges whose weight is less than the 'edge threshold' are removed from the graph. For example, if the edge threshold is 2 (the default), the connection is broken between any two sequences that share only a single motif.

The display shows a thumbnail of each connected component, suppressing figures for connected components that consist of only one sequence. These thumbnails can be expanded into larger figures, including EPS output for printing or publication. The graphs are visualized using the random layout option in NetworkX [13], which we found to be both the fastest and most readable option for the highly connected graphs produced by MotifCluster.

### Implementation

Most code described here was written in Python 2.4 and tested on MacOSX and Linux. The exceptions are the NW algorithm [10], which we implemented in C for performance reasons, the MUSCLE [12], MEME [5] and Gibbs Sampler [6] programs, and the libstree suffix tree library [14], for which we used the published implementations. The web interface uses Apache and mod_python (Apache Software Foundation). Motif clustering jobs are submitted to our Beowulf cluster using PBS/TORQUE. NetworkX [13] is used in the graph calculations. PyMol [9] is used for visualization of protein structures. Calculation and display code have been contributed to the PyCogent project [15]. A standalone version of the program is available for download at the MotifCluster web site.

## Example analyses

We describe the capabilities of MotifCluster using four cases as examples. First, we use the case of the two convergently evolved families of ribose 5-phosphate isomerases to show that structurally distinct proteins that have the same function can be correctly clustered. Second, we use a set of curated superfamilies that contains 4,887 sequences in 91 families divided among 5 superfamilies [16]. These 'gold-standard' families and superfamilies allow us to test how well we can recapture known relationships within and between superfamilies. Third, we use two families within the haloacid dehalogenase superfamily [17] to illustrate the utility of the clustering and mapping features of MotifCluster. Finally, we show how clustering of motifs in a set of proteins from the highly divergent thioredoxin-fold suprafamily [7] captures evolutionary relationships between proteins of different functions when standard phylogenetic analyses fail.

### MotifCluster distinguishes between unrelated families when the edge threshold is 2 or greater: RpiA/RpiB as a case study

Ribose 5-phosphate isomerases catalyze the interconversion of ribulose 5-phosphate and ribose 5-phosphate. Two structurally distinct families of ribose 5-phosphate isomerases have been identified, exemplified by RpiA from *Escherichia coli *and RpiB from *E. coli *[18,19]. This is one of many cases of convergent evolution of the same catalytic activity in the context of different structural folds. It is unlikely that the same motifs would evolve in different structural contexts. Thus, a potential application of MotifCluster is the identification of unrelated families in sets of proteins that have a common function. In such cases, clustering of sequences into two or more families does not constitute evidence of convergent evolution in the absence of structural information, but it raises a possibility that can be further investigated.

**Figure 2:**
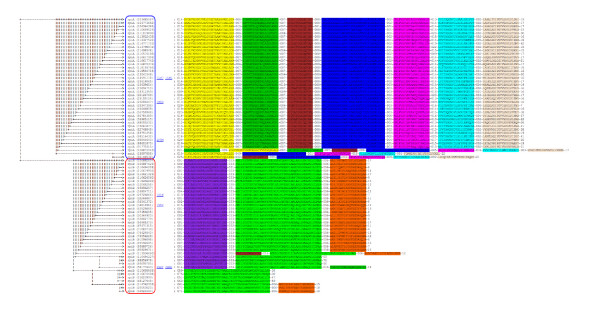
Clustering of motifs found in 80 members of the RpiA and RpiB families of ribose 5-phosphate isomerases. The blue box encloses RpiAs, and the red box encloses RpiBs.

RpiA sequences were found using PSI-BLAST [2] with an E-value of 10^-10 ^and an H-value of 10^-20 ^with *E. coli *ribose 5-phosphate isomerase (gi 16130815) as the seed. A divergent set of 41 sequences was picked from the 465 sequences found by PSI-BLAST using DivergentSet [20] with a 55% identity threshold cutoff. These sequences range from 218-271 residues in length (average 235) and have an average pairwise identity of 47.4%. RpiB sequences were found using PSI-BLAST with an E-value of 10^-10 ^and an H-value of 10^-20 ^with *E. coli *ribose 5-phosphate isomerase B (gi 16131916) as the seed. A divergent set of 39 sequences was chosen from the 412 sequences found by PSI-BLAST search using a 55% identity threshold cutoff. The RpiB sequences range from 140-187 residues in length (average 153) and have an average pairwise identity of 46.4%. Motifs in the combined set of sequences were found by MEME using an E-value threshold of 10^-20 ^and a setting of 10 expected motifs. Figure 2 shows a clustering of these 80 sequences based on the motifs they contain, using the NW module alignment 1-(actual/max) distance metric with weighted motifs. Sequences of RpiA homologs are circled in red, and sequences of RpiB homologs are circled in blue. The two families fall into two separate components when an edge threshold of 2 is chosen. A similar result is achieved even if the sequences in the set are not pre-ordered into related groups. Note that several sequences in the set lack one or more motifs characteristic of the family, but no sequence is incorrectly placed into the wrong family. If an edge threshold of 1 is chosen, a single false-positive motif connects the two families into one component.

### Analysis of the gold-standard superfamilies shows that the sensitivity and specificity of MotifCluster are excellent

We tested MotifCluster on the gold-standard set of mechanistically diverse superfamilies described by Brown *et al*. [16], which contains 4,887 sequences belonging to 91 families, distributed among 5 superfamilies. This set of sequences has been carefully curated to provide a reliably clustered set for testing computational algorithms. Every sequence assigned to a gold-standard family has either an experimentally determined function, or is closely related to a protein of known function (BLAST e-value ≤ 10^-175^). We tested MotifCluster using different edge threshold settings (that is, the number of motifs required for a shared connection), and using the Gibbs sampler and MEME to find the underlying motifs, in order to test how well it was able to cluster family members within the same superfamily. Specifically, we expect sequences from the same superfamily to be connected by multiple motifs, but we do not expect members of different superfamilies to be bridged in this manner.

**Figure 3:**
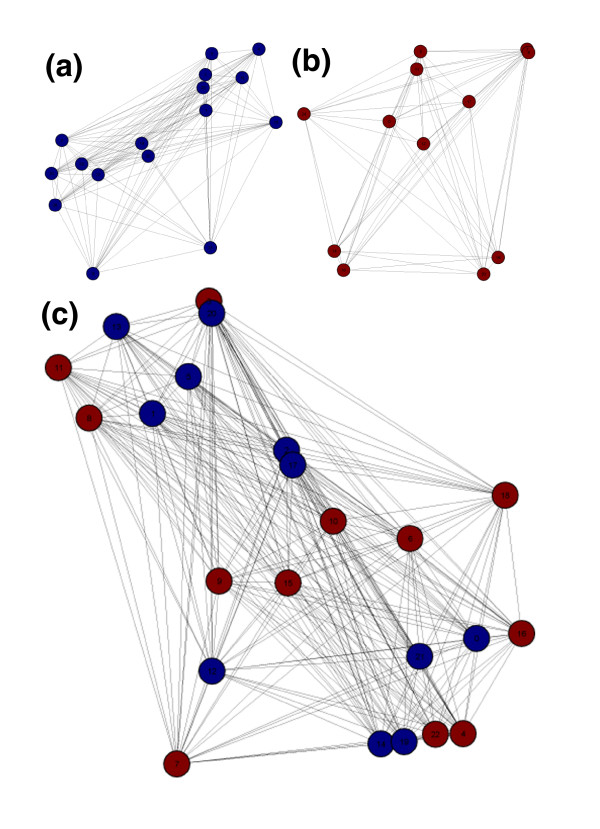
Graph representation of clusters generated from motifs identified in **(a) **members of the dihydroorotase and **(b) **β-phosphoglucomutase families, which belong to separate superfamilies, and **(c) **members of the 2-haloacid dehalogenase (blue) and β-phosphoglucomutase (red) families, which belong to the same superfamily. The families can be subdivided further into additional groups by increasing the edge threshold.

Figure 3a shows graphs of connected components generated from sequences representing two distinct superfamilies, using an edge threshold of 2. Dihydroorotases (red) belong to the amidohydrolase superfamily, and β-phosphoglucomutases (blue) to the haloacid dehalogenase superfamily. The sequences form two connected components, as expected because only families within the same superfamily should share homologous motifs. Families from different superfamilies should not be connected, except when the significance threshold is so low that motifs are found by chance.

Figure 3b shows a graph of a single connected component that contains sequences from two different families belonging to the amidohydrolase superfamily. Haloacid dehalogenase (blue) and β-phosphoglucomutase (red) are divergent members of the haloacid dehalogenase superfamily (Figure 3c). The maximum pairwise identity between members of the two families is 45.5%. When an edge threshold of 3 or less is used, all of the sequences are grouped into a single connected component.

**Figure 4:**
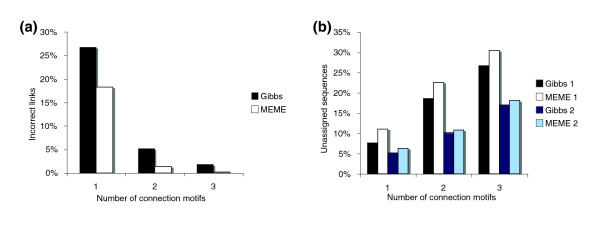
Summary of the performance of MotifCluster using motifs found by MEME and the Gibbs sampler for 741 pairs of families in the gold-standard set of families. **(a) **Incorrect inferences of superfamily assignment. **(b) **Failure to assign sequences to the leading component (for members of the same superfamily) or to one of the two leading components (for members of two different superfamilies). The numbers 1 and 2 in the legend (for example, Gibbs 1 and Gibbs 2) refer to the two largest components, which invariably contain most of the sequences from the two distinct families when these families belong to different superfamilies.

Statistics describing the performance of MotifCluster in analyses of all pairs of the 91 sequence families described by Brown *et al*. (a total of 4,186 pairs) are given in Figure 4. In each case, motifs were found using a combined set of the reference sequences from the two families by both MEME (using the following parameters: -protein -minw 8 -maxw 40 -nmotifs 10 -evt 1e-5 -mod anr -maxsize 14173) and the Gibbs sampler (using the following command-line parameters: 14,16,18,20,22,24,26,28,30 10,10,10,10,10,10,10,10,10 -W 0.8 -w 0.1 -p 45 -j 5 -i 500 -S 20 -C 0.5). In this analysis, the false positive rate is defined as the rate at which a link is incorrectly inferred between two families from different superfamilies, and the false negative rate is defined as the rate at which a link between two families from the same superfamily is missed. Using motifs found by MEME, the false positive rate was 1.3% using an edge threshold of two, and 0.17% using an edge threshold of three. The corresponding figures for analyses using motifs found by the Gibbs sampler were 5.2% and 1.8%, respectively. The false positive rate using an edge threshold of one was always high (18% and 27% for MEME and Gibbs, respectively), suggesting that the shared presence of a single motif is insufficient for the inference of homology between two families. This result is expected, as a single false-positive motif occurrence in any member of the set will join the two components into a single cluster.

The false negative rate in this analysis was essentially zero (data not shown). No false negatives were found at all except when an edge threshold of three was used for motifs found by MEME. In that case, the algorithm failed to find a link between the deoxy-D-mannose-octulosonate 8-phosphate phosphatase and P-type ATPase families in the haloacid dehalogenase superfamily. Thus, the presence of at least two shared MEME motifs is a strong indicator of shared superfamily membership, whereas absence of at least two shared MEME motifs is a strong indicator of lack of shared superfamily membership. However, failure to assign all sequences to a single cluster (for members of the same superfamily) or to two distinct clusters (for members of different superfamilies) is frequent (Figure 4b). The average fraction of unassigned sequences ranged from 5.2% (Gibbs sampler, edge threshold of 1, two superfamilies) to 30.4% (MEME, edge threshold of 3, one superfamily), showing that not all family members share motifs (at least, as defined by MEME or the Gibbs sampler), even when homology exists at the primary sequence level. Thus, the presence or absence of shared motifs at the whole family level is informative, but the fact that individual sequences lack motifs shared by the other sequences in the set does not indicate that they are not homologous. The error rates were robust to variation in the degree of divergence between the sequences (average pairwise identities between the families ranged from 38.4-57.9%), the number of sequences in each family (which ranged from 5-366), and differences between the sample size in the two families (the fraction of sequences represented by one of the two families ranged from 0.014-1). No significant correlations were observed between these variables and false positive rate, false discovery rate, false negative rate, sensitivity, or specificity (data not shown).

### MotifCluster facilitates identification of conserved and variable residues in active sites of mechanistically divergent families

**Figure 5:**
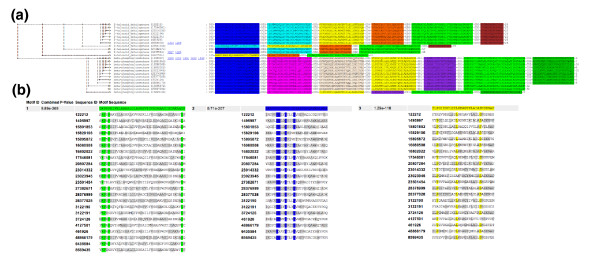
Analysis of haloacid dehalogenases. **(a) **Clustering of motifs in the haloacid dehalogenase and β-phosphoglucomutase families of the haloacid dehalogenase superfamily. **(b) **Sequences of the three shared motifs, with highly conserved and mechanistically important residues highlighted by MotifCluster.

**Figure 6:**
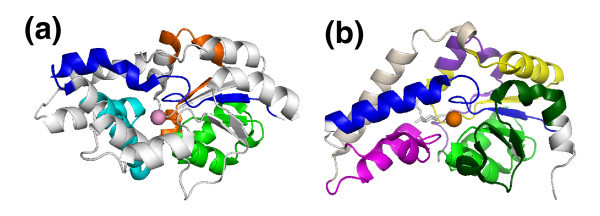
Motifs identified by MEME mapped onto the crystal structures of **(a) **haloacid dehalogenase [PDB:1QQ7] and **(b) **β-phosphoglucomutase [PDB:1O03] by MotifCluster.

**Figure 7:**
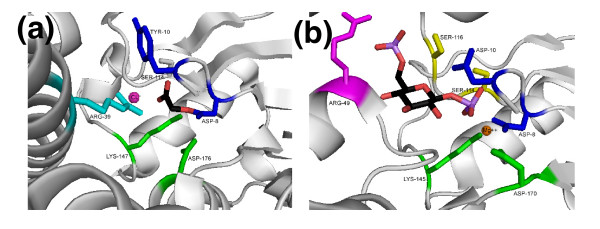
Active site regions of **(a) **haloacid dehalogenase and **(b) **β-phosphoglucomutase, with conserved residues highlighted according to the motif color scheme shown in Figure 6. Note that the side-chain coloring was added manually in PyMol.

Figure 3c shows that sequences in the haloacid dehalogenase and β-phosphoglucomutase families can be clustered into a single connected component by MotifCluster, consistent with the known evolutionary relationship between these families [16]. Although the reactions catalyzed by the prototypical members of these two families are quite different, each reaction involves attack of a nucleophilic Asp residue in the initial step of the reaction. The reactions differ, though, in the nature of the atom attacked by the Asp, the mechanism for stabilization of the leaving group, and the requirement for Mg^2+ ^in the β-phosphoglucomutases. Figure 5a shows the motifs characteristic of the two families; notably, three motifs are found in most members of both families, suggesting that these represent regions of the protein responsible for conserved functions. Within these three motifs, certain positions stand out as being conserved in both families, or in only one family (Figure 5b). MotifCluster facilitates analysis of evolutionary relationships among protein families by automatically mapping motifs onto the structures of structurally characterized members of the set (Figure 6). The blue and light green motifs contribute to the active site in both proteins. Zooming into the active site structures in PyMol (Figure 7) shows that the nucleophilic Asp residue occupies a comparable position in both structures. Notably, two residues in the green motif (a Lys and an Asp) are structurally conserved, but play different roles in the two enzymes. In the β-phosphoglucomutase, Lys145 forms a salt bridge to the phosphate of the substrate. In the haloacid dehalogenase, the comparable residue (Lys147) forms a salt bridge to the terminal carboxylate of the haloacid substrate. In the β-phosphoglucomutase, Asp170 coordinates the active site Mg^++^. The comparable residue in the haloacid dehalogenase (Asp176) forms a hydrogen bond with Lys147. In addition, an active site Ser forms a hydrogen bond to the substrate in both cases. Residues in the other motifs that are conserved only in one of the two families are identifiable in the active site, as well; these residues contribute to family-specific functions such as stabilization of the chloride leaving group in the haloacid dehalogenase family. This type of analysis has traditionally been carried out by manual mapping of motifs discovered by MEME or the Gibbs sampler onto structures in a separate structure visualization package. By automating this process, MotifCluster speeds up the analysis and allows rapid analysis of multiple sets of different composition, which can be important because the motifs found by MEME and the Gibbs sampler vary somewhat according to the composition of the input set.

### The various clustering methods available in MotifCluster facilitate analysis of extremely distantly related families

The Trx-fold suprafamily encompasses an extremely divergent set of proteins with a wide range of functions. All members of the suprafamily share the canonical Trx-fold structure, but the ancestral function (reduction of disulfide bonds using a pair of active site cysteines) has been modified in some superfamilies. For example, in the peroxiredoxin family, a cysteine corresponding to the more buried cysteine in Trxs is involved in reduction of peroxides, but the other cysteine has been changed to a threonine [7]. In the glutathione transferase superfamily, both cysteines have been lost, and these enzymes catalyze a completely different reaction: nucleophilic attack of glutathione upon an electrophilic substrate to form a glutathione conjugate. Analysis of sequence relationships among such highly divergent proteins is difficult because the overall pairwise sequence identities fall within the twilight zone. In such cases, identification of shared motifs in proteins that share a common structural fold can provide good evidence for a very distant evolutionary relationship.

An analysis of the relationship between thioredoxins (Trxs) and peroxiredoxin (Prxs) was reported in 2004: no significant pairwise identity could be demonstrated between sequences in these two families, but the Shotgun algorithm [3] identified a family of proteins, the cytochrome maturation proteins (CMPs), that bridges the Trxs and Prxs [7]. Motifs found in subsets of these three families were identified by MEME. The results were clustered manually, a time-consuming and qualitative process. MotifCluster performs a comparable analysis in a few minutes. Here we demonstrate that the use of different distance metrics produces different clustering results. Notably, clustering using motifs rather than whole sequences produces biologically meaningful results even when standard phylogenetic clustering methods fail due to the extremely divergent set of proteins in the analysis.

**Figure 8:**
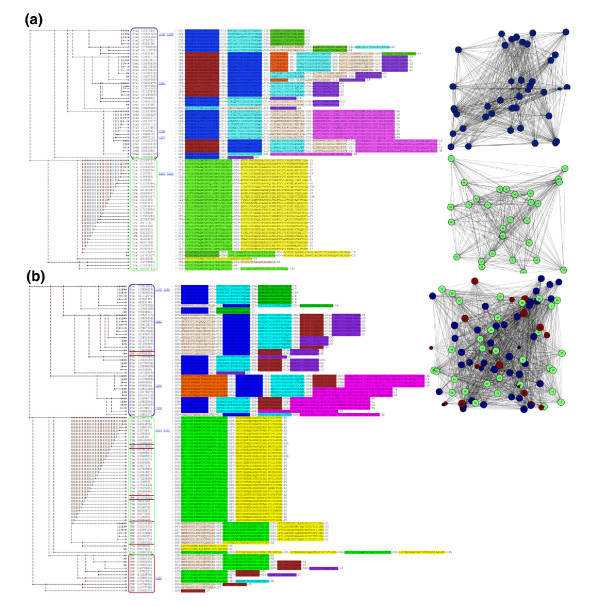
Clustering of a divergent set of 96 sequences from the Prx, Trx and CMP families. Prxs are circled in red, Trxs are circled in blue and CMPs are circled in green. In each case, both the clustering of motifs and the connected components are shown. **(a) **Clustering of the Prx (top right graph) and Trx (bottom right graph) families using the NW module alignment 1-(actual/max) score; **(b) **clustering of the Prx, Trx, and CMP families using the NW module alignment 1-(actual/max) score. The 34 Trx sequences range from 89-578 residues in length (average 141) and are 48.6% identical on average. The 40 Prx sequences range from 133-321 residues in length (average 180) and are 43.8% identical on average. The 22 CMP sequences range from 121-403 residues in length (average 194) and are 44.7% identical on average.

**Figure 9:**
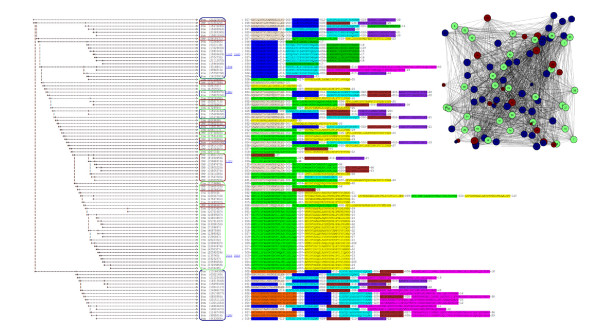
Phylogenetic tree of 96 sequences from the Prx, Trx and CMP families. This figure shows clustering of the Prx, Trx, and Cmp families using the phylogenetic tree generated by MUSCLE. See legend to Figure 8 for details of the sequence families and display.

Figure 8a shows that, in the absence of the bridging CMP sequences, the Trx and Prx families cluster into two components, indicating that no evolutionary relationship can be discerned. (Trxs are circled in blue and Prxs in red.) When motifs are found using a set of 96 proteins representing Trxs, CMPs and Prxs [7], MotifCluster clusters the proteins into a single connected component (Figure 8b) using an edge threshold of 2 and the NW module alignment 1-(actual/max) score, which considers only the sequences of the motifs. However, clustering using the phylogenetic tree generated using MUSCLE is quite poor (Figure 9). This poor performance is expected because of the high level of sequence divergence. On this data set, the other distance metrics give results of intermediate quality, but the motif-based clustering is always better than the phylogenetic clustering.

## Implications for motif analyses

Motif identification informs functional, mechanistic and evolutionary analyses in several ways. First, the patterns of motifs observed in subsets of the input set can be used to cluster the proteins into families, a useful tool for prediction of function for unannotated proteins. Second, motifs indicate regions of proteins that have been conserved for reasons of structure and/or function. Changes in a region of a protein family, either resulting in a different motif or in subtle, family-specific changes within a motif, suggest the changes that have led to emergence of new functions in an ancestral scaffold.

MotifCluster takes input from motif-finding algorithms such as MEME or the Gibbs sampler, and the results are therefore dependent upon the choice of the input set because the characteristics of the input set have a strong effect upon the motifs that are found. A crucial limitation of existing techniques is that motif-finding algorithms typically assume that each sequence is drawn independently from a background distribution, and thus can be biased by the presence of closely related sequences. We previously addressed this problem with our software DivergentSet [20], which allows the user to rapidly select an unbiased sample of divergent sequences from the starting population. In several sequence families, our analysis of different divergent sets drawn repeatedly from each family showed that the motifs found by MEME were highly dependent on the particular set chosen: the number of motifs, and the lengths and locations of the motifs, varied from run to run [20]. Although highly conserved motifs that are critical for function are reliably recovered, the robustness of any motif analysis is enhanced when multiple analyses can be carried out using divergent sets generated randomly from a larger set of homologous sequences.

Analysis of the importance of sequence motifs is greatly enhanced when motifs can be mapped onto the structure of representative proteins. Such mapping allows visualization of motifs that are found in the core of the protein and may be responsible for maintaining the overall structural fold, motifs that are on the surface and may be involved in interactions with ligands or other proteins, and motifs that are found in clefts or crevices that may harbor active sites. The automation of the clustering of sequences and the mapping of motifs onto structures substantially reduces the time required to carry out such analyses, and will provide many new insights into the relationships among highly divergent protein families.

## Abbreviations

CMP: cytochrome maturation protein; IDs: identifiers; LCS: Longest common substring; MEME: Multiple EM for motif elicitation; MUSCLE: Multiple sequence comparison by log-expectation; NW: Needleman-Wunsch; PDB: Protein Data Bank; Prx: peroxiredoxin; Trx: thioredoxin; UPGMA: Unweighted pair group method with arithmetic mean.

## Authors' contributions

MH and JW developed the software. SDC and RK directed the research. RK and MH wrote the manuscript, with most figures provided by JW.
